# Tenofovir and adefovir down-regulate mitochondrial chaperone TRAP1 and succinate dehydrogenase subunit B to metabolically reprogram glucose metabolism and induce nephrotoxicity

**DOI:** 10.1038/srep46344

**Published:** 2017-04-11

**Authors:** Xinbin Zhao, Kun Sun, Zhou Lan, Wenxin Song, Lili Cheng, Wenna Chi, Jing Chen, Yi Huo, Lina Xu, Xiaohui Liu, Haiteng Deng, Julie A. Siegenthaler, Ligong Chen

**Affiliations:** 1 School of Pharmaceutical Sciences, Tsinghua University, Beijing, 100084, China; 2 Collaborative Innovation Center for Biotherapy, State Key Laboratory of Biotherapy and Cancer Center, West China Hospital, West China Medical School, Sichuan University, Chengdu, 610041, China; 3 MOE Key Laboratory of Bioinformatics, School of Life Sciences, Tsinghua University, Beijing, 100084, China; 4 Technology Center for Protein Sciences, School of Life Sciences, Tsinghua University, Beijing, 100084, China; 5 Department of Pediatrics, Denver-Anschutz Medical Campus, University of Colorado, Aurora, CO 80045, USA

## Abstract

Despite the therapeutic success of tenofovir (TFV) for treatment of HIV-1 infection, numerous cases of nephrotoxicity have been reported. Mitochondrial toxicity has been purported as the major target of TFV-associated renal tubulopathy but the underlying molecular mechanism remains unclear. In this report, we use metabolomics and proteomics with HK-2 cells and animal models to dissect the molecular pathways underlying nephropathy caused by TFV and its more toxic analog, adefovir (ADV). Proteomic analysis shows that mitochondrial chaperone TRAP1 and mtDNA replicating protein SSBP1 were significantly down-regulated in TFV and ADV treated HK-2 cells compared with controls. Transmission electron microscopy (TEM) revealed that TFV and ADV-treated HK-2 cells had accumulated glycogen, a phenotype that was also observed in mice treated with TFV and ADV. Analysis of the proteins in TCA cycle showed succinate dehydrogenase subunit B (SDHB) was nearly depleted in glucose oxidative phosphorylation pathway however certain enzymes in the glycolysis and glycogen synthesis pathway had elevated expression in TFV and ADV-treated HK-2 cells. These results suggest that TFV and ADV may cause mitochondrial dysfunction in renal tubular cells and reprogramming of glucose metabolism. The resulting glycogen accumulation may partially contribute to TFV and ADV induced renal dysfunction.

Tenofovir (TFV) has been extensively used worldwide for long-term treatment of human immunodeficiency virus (HIV) and chronic hepatitis B viral (HBV) infections^1^,^2^,^3^. Although TFV has favorable efficacy, safety and low resistance profiles compared with other antiretroviral drugs^4^,^5^,^6^, numerous cases have been published describing acute kidney injury, tubulopathies, nephrolithiasis, chronic kidney disease and Fanconi syndrome with long-term TFV use^7^,^8^,^9^. TFV undergoes elimination from systemic circulation via a combination of glomerular filtration and active tubular secretion^10^,^11^. TFV can be taken up by human organic anion transporter 1 (hOAT1) and hOAT3 and efflux by the multidrug resistance protein (MRP4), respectively^11^,^12^,^13^,^14^. The proximal tubule is suggested as the main site of TFV-associated toxicity however the underlying mechanisms are not well understood^15^. Nucleoside reverse transcriptase inhibitors (NRTIs) are effective antivirals due to their high affinity for the viral DNA polymerase, reverse transcriptase^15^,^16^. NRTIs can also inhibit structurally similar human DNA polymerase γ (POLG), the enzyme responsible for replication of mitochondrial DNA (mtDNA)^17^,^18^. Thus, mitochondrial toxicity has been postulated as the major target of NRTIs-associated renal tubular disease^4^,^5^,^19^. However TFV is a weaker inhibitor of polymerase γ than most of the other NRTIs and incorporation of TFV into a DNA primer-template by DNA polymerase γ is also less efficient. Therefore it is possible that TFV acts on a pathway or cell function other than mtDNA synthesis to induce nephropathy.

Most studies of renal toxicity caused by TFV and ADV use focus on the clinical outcomes and parameters monitoring kidney injury. There are few mechanistic studies thus the mode of action behind TFV and ADV-associated renal toxicity remains to be fully understood. To elucidate the mechanisms of TFV and ADV induced renal toxicity, we selected human renal proximal tubule cell (HK-2) and mouse renal proximal tubular epithelial cells (RTEC). Following treatments of HK-2 cells with TFV and ADV at various conditions, we systematically investigated the phenotypes and molecular pathways with metabolomics and proteomics. Furthermore, we treated mice with tenofovir disoproxil fumarate (TDF) and adefovir dipivoxil (DPADV), as oral prodrugs of TFV and ADV, for 8 weeks to evaluate their kidney toxicity and the molecular pathways proved in the cellular study. Based on our observations and molecular analyses *in vitro* and *in vivo*, we propose glycogen accumulation caused by reprograming of glucose pathways as a novel mechanism contributing to the TFV and ADV associated nephrotoxicity.

## Results

### Evaluation of TFV and ADV’s cytotoxicity in HK-2 and RTEC

**Figure 1:**
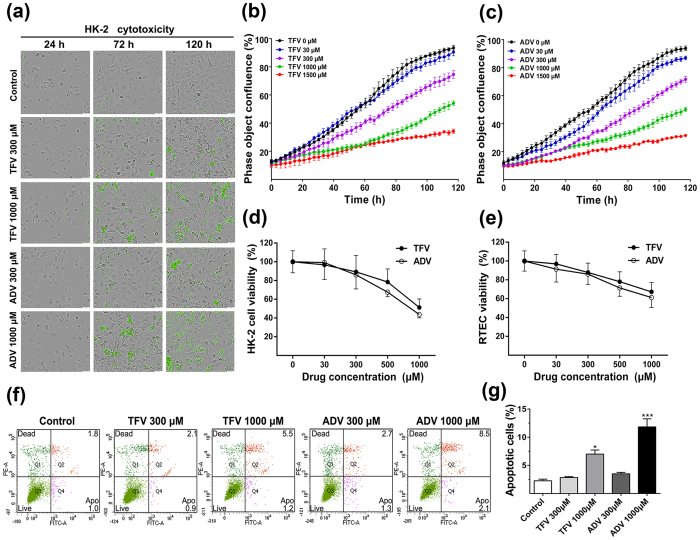
Effects of TFV and ADV on cell growth, proliferation and toxicity. (**a**) HK-2 cells were treated with TFV and ADV at various concentrations for 5 days. Phase-contrast and fluorescent images were observed. (**b**,**c**) The inhibition of cell proliferation was calculated by dividing the number of YOYO^®^-1 fluorescent objects (cytotoxicity index) by the total number of objects. (**d**,**e**) Dose- and time- dependent cytotoxicity of TFV and ADV after incubation of HK-2 cells and RTEC. Cell viability was determined by MTT assay. (**f**) HK-2 cells treated with 0, 300, 1000 μM of TFV and ADV for 5 days were stained with Annexin-V/PI for FACS-based quantification of apoptotic cells. (**g**) Quantification of apoptotic cells in HK-2 cells per group. All results are presented as means ± SEM (N = 3, *P < 0.05, ***P < 0.001 vs control).

In order to examine the effects of TFV and ADV on cell growth and toxicity, we treated HK-2 cells with TFV and ADV at various concentrations for 5 days then used the IncuCyte™ ZOOM basic analyzer to quantify the cell proliferation rate for *in vitro* cytotoxicity. Increased green fluorescence, indicative of membrane integrity loss, was most pronounced in cells treated with a high concentration (1000 μM) while some nuclear labeling indicative of dead/dying cells was observed at 300 μM (Fig. 1a)^20^. TFV and ADV showed apparent inhibition on HK-2 cells growth (about 50% for both drugs at 1000 μM), indicating that TFV and ADV are tolerated but cytotoxicity may occur at high concentrations (Fig. 1b,c). To further evaluate the cytotoxicity of TFV and ADV, MTT assay was applied in HK-2 cells and RTEC (Fig. 1d,e). TFV and ADV treatment significantly decreased cell viability (55% for HK-2 and 40% for RTEC at 1000 μM). At 1500 μM of TFV or ADV, viability was severely compromised in HK-2 cells with about 65% and 70% death, respectively (Fig. 1b,c). Though 1000 μM of TFV or ADV significantly decreased cell viability (Fig. 1b,c), sufficient live cells remained for experimentation. Thus, for subsequent studies we used 5-day and 1000 μM for TFV and ADV as the end-point treatment conditions to induce toxicity and cellular alternations.

To examine the effect of TFV and ADV on apoptosis, HK-2 cells were stained with Annexin-V/PI. Annexin V^+^/PI^−^ cells represent the early apoptotic populations and Annexin V^+^/PI^+^ cells represent late apoptotic populations. The cells are incubated with Annexin V prior to harvesting, thus apoptotic cells (Annexin V^+^/PI^−^ and Annexin V^+^/PI^+^) can be distinguished from cells damaged during isolation (Annexin V^−^/PI^+^). Percentages of apoptotic populations in each treatment were determined by flow cytometry (Fig. 1f). At 300 μM, TFV and ADV did not significantly increase apoptosis. However, the number of apoptotic cells (Annexin V^+^/PI^−^ and Annexin V^+^/PI^+^) increased to approximately 8% and 13% following 1000 μM of TFV and ADV treatments, respectively (Fig. 1g). These observations further support HK-2 cells as an appropriate cell model for testing the mechanisms underlying cytotoxicity caused by TFV and ADV treatment.

### Characterization of TFV and ADV induced mitochondrial and glycolytic dysfunction

**Figure 2:**
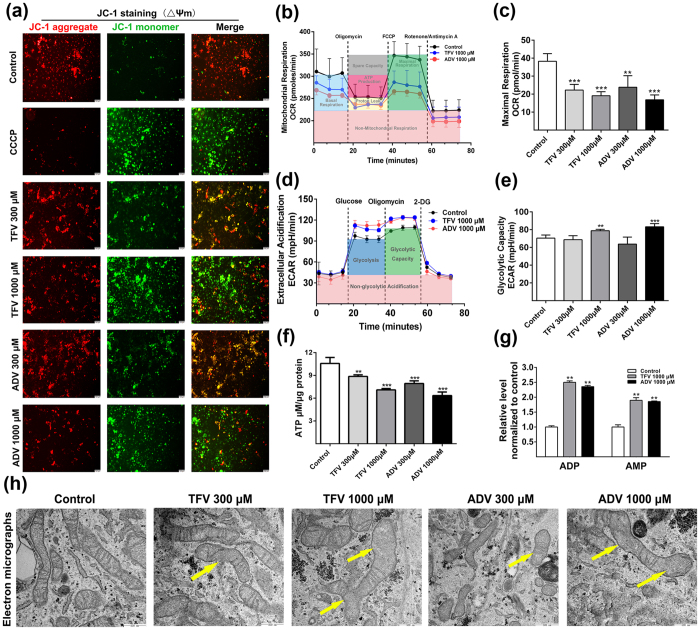
Effect of TFV and ADV on mitochondrial and glycolytic function in HK-2 cells. (**a**) HK-2 cells were treated as previously described for measuring mitochondrial membrane permeability. The cells were stained with JC-1. Red (JC-1 aggregate)/green (JC-1 monomer) fluorescence intensity represents the potential alteration of mitochondria membrane. (**b**) After 2-day treatments, cells were measured by the mitochondrial stress test kit to determine the oxygen consumption rate. Representative mean OCR traces at baseline following injections of reagents. (**c**) Maximum respiration were calculated from the mean OCRs. (**d**) Glycolysis stress test was used to measure glycolytic function of HK-2. Representative mean ECAR traces at baseline following injections of reagents. (**e**) Glycolytic capacity were calculated from the mean ECARs. (**f**) ATP levels in HK-2 cells by ATP determination assay. (**g**) ADP and AMP levels in HK-2 cells treated by TFV and ADV at 1000 μM were measured by metabolomic assay. (**h**) Representative electron micrographs of HK-2 cells treated with or without TFV and ADV. TFV and ADV treatments caused irregular mitochondrial shape and fragmented cristae (yellow arrows) (original magnification 60000x, marker indicates 500 nm). Values are presented as means ± SEM (N = 3, *P < 0.05, **P < 0.01, ***P < 0.001 vs control).

Mitochondrial disruption has been proposed as the mechanism underlying TFV and ADV induced renal toxicity^18^. To characterize the effect of TFV and ADV on mitochondria, we conducted a series of assays for mitochondria membrane potential (MMP), respiration and morphology (Fig. 2).

To evaluate the effect of TFV and ADV on the MMP in HK-2 cells, we examined the fluorescent intensities of retained JC-1 aggregate (red) and leaked JC-1 monomer (green) as an indication of changes in MMP. Carbonyl cyanide m-chlorophenylhydrazone (CCCP) causes quick mitochondrial membrane depolarization to provide a strong positive green-signal fluorescence control. Qualitatively, TFV and ADV treatments reduce MMP as indicated by decreased red JC-1 aggregates and increased green JC-1 monomers with the effect most pronounced at 1000 μM (Fig. 2a), suggesting that TFV and ADV disrupt MMP in HK-2 cells and cause mitochondrial dysfunction.

To assess TFV and ADV’s effect on oxygen consumption rate (OCR) of mitochondrial respiration and extracellular acidification rate (ECAR) of glycolysis, we used Seahorse XF analyzer to measure mitochondria and glycolysis function. Figure 2b depicts OCR traces of HK-2 cells exposed to TFV and ADV (1000 μM). Maximal respiration was examined by assessing uncoupled OCR as an indicator of mitochondrial dysfunction. Maximal respiration significantly decreased ~50% following 300 or 1000 μM TFV and ADV treatments as compared with controls (Fig. 2c). Accordingly, ATP produced by the mitochondria declined to about half of the untreated group (Supplementary Fig. 1a). Proton leak was significantly decreased in TFV and ADV treatments (Supplementary Fig. 1b). Proton leak has important functions in the coupling of ATP synthesis to oxygen consumption therefore reduced proton leak following TFV and ADV treatments might increase the ATP production in these cells experiencing ATP short fall. However as the basal respiration of mitochondria was lower than that of controls (Supplementary Fig. 1d), the increased ATP from proton leak was unlikely to fully compensate for overall mitochondrial damage. The spare capacity (calculated as maximum respiration OCR-basal respiration) was also significantly reduced to more than 50% and 75% compared with the controls following treatments, respectively (Supplementary Fig. 1c). The general scheme of glycolysis stress test of HK-2 cells treated with TFV and ADV (300, 1000 μM) was shown in Fig. 2d. Glycolytic capacity was significantly increased after oligomycin injection in TFV and ADV-treated groups (1000 μM) compared with the controls (Fig. 2e). Collectively, these data demonstrate that TFV and ADV treatment significantly reduce mitochondrial respiration rates but enhance glycolysis in HK-2 cells.

We next quantified ATP content using an ATP determination kit. There was a significant decrease in ATP production in HK-2 cells treated with 300, 1000 μM of TFV and ADV (Fig. 2f). Furthermore, in our metabolomics assay of HK-2 cells with TFV and ADV (1000 μM), Adenosine diphosphate (ADP) and adenosine monophosphate (AMP) levels were elevated about 2.5-fold and 2-fold higher than the controls, respectively (Fig. 2g, Supplementary Table 1). ADP and AMP are two intermediates in energy transfer and biosynthetically synthesized by various pathways^21^ therefore the increase in ADP and AMP is likely only due in part to decreased ATP synthesis. This data suggests TFV and ADV treatment reduces ATP production resulting in lower cellular energy status and is consistent with the altered mitochondria respiration described in Fig. 2b. We next used transmission electron microscopy (TEM) to examine TFV and ADV’s effect on mitochondrial ultrastructure. At 300 μM TFV or ADV, HK-2 cells showed irregular shaped mitochondria and disrupted cristae and these changes (yellow arrows) were more pronounced at 1000 μM (Fig. 2h, Supplementary Fig. 2a,b) implying that disruption of electron transport chain and ATP production occurred^22^,^23^. This mitochondrial damage is likely a major underlying cause of diminished mitochondrial respiration and ATP production observed with TFV and ADV treatment.

### Proteomic profiles of HK-2 cell treated with TFV and ADV

**Figure 3:**
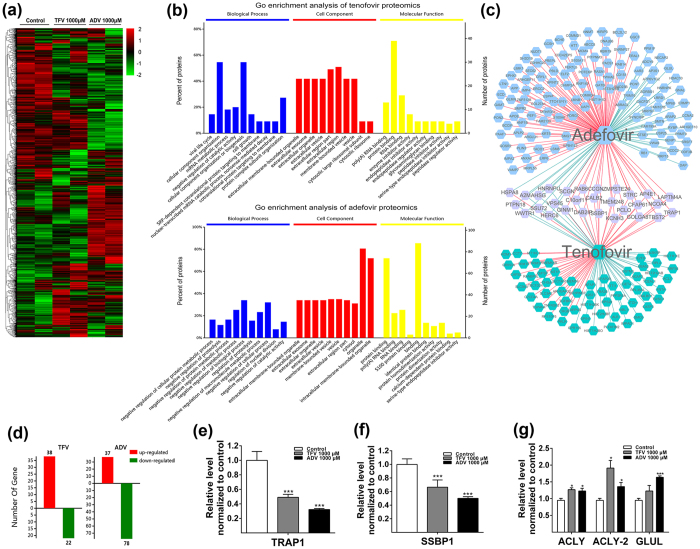
Analysis of proteomic data for HK-2 cells treated with TFV and ADV at 1000 μM. (**a**) Heat map of significantly changed proteins following TFV and ADV treatments on HK-2 cells. (**b**) GO enrichment analysis for biological process, cell component and molecular function in HK-2 after TFV and ADV treatments. (**c**) The overlapped proteins between TFV and ADV treatments analyzed above. (**d**) Analysis of up- or down-regulated proteins with at least two unique peptides. (**e**) Ratio of TRAP1 in TFV and ADV treatments over controls from proteomics data. (**f**) Ratio of SSBP1 in TFV and ADV treatments over controls from proteomic data. (**g**) Significantly regulated bioenergetic proteins ACLY and GLUL ratios in TFV and ADV treatments over controls from proteomic data. Ratio was calculated as: T1/C1, T2/C1, T1/C2 and T2/C2 for TFV; A1/C1, A2/C1, A1/C2 and A2/C2 for ADV. *P < 0.05, ***P < 0.001.

To identify cellular pathways effects by drug treatment, we used proteomic profiling to identify changes in protein expression that occur in TFV and ADV-treated HK-2 cells. A total of 4697 proteins were identified by LC-MS/MS. The quantitative ratios compared between TFV, ADV and control groups are depicted in a heat map (Fig. 3a). The proteomic data was further analyzed by Gene Ontology and KEGG pathway analysis to subdivide protein changes into three broad categories, biological process, cellular component and molecular function (Fig. 3b). Comparison analysis to identify significantly altered peptides revealed 38 up-regulated proteins and 22 down-regulated proteins following TFV treatment and 37 up-regulated proteins and 78 down-regulated proteins following ADV treatment (Fig. 3d). Furthermore, we identified 24 proteins that changed consistently in TFV and ADV compared with controls (Fig. 3c,d).

Our proteomic data of HK-2 cells with TFV and ADV treatments identified TNF receptor-associated protein 1 (TRAP1), previously shown to play a critical role in energy homeostasis and glucose metabolism^24^, as the most down-regulated protein. The mean ratio for TRAP1 was significantly decreased to 0.491 and 0.323 after TFV and ADV treatments, respectively (Fig. 3e). The protein-protein interactome for TRAP1 with other proteins in the proteomics dataset were generated using String 10 database^25^ and then imported into Cytoscape software^26^ with differentially expressed proteins marked in red (supplementary Fig. 1e). Within the list of down-regulated proteins (Supplementary Table 2), we found the mean ratios of single-stranded DNA-binding protein (SSBP1) were decreased 0.665 for TFV and 0.500 for ADV (Fig. 3f). SSBP1 was in the top 5% of down-regulated proteins in the proteomic data set and has been shown to be important for mitochondrial DNA replication^27^. The protein-protein interaction network of SSBP1 is shown in Supplementary Fig. 1f from String 10 database and Cytoscape software. We also identified significant up-regulation in proteins involved in fatty acid or glutamine metabolism, ATP-citrate synthase (ACLY), isoform 2 of ACLY and glutamine synthetase (GLUL) (Fig. 3g, Supplementary Table 2). These proteins may also have important roles in the molecular mechanism of TFV and ADV associated renal toxicity.

### Effect of TFV and ADV on SSBP1 involved in mitochondrial DNA replication

**Figure 4:**
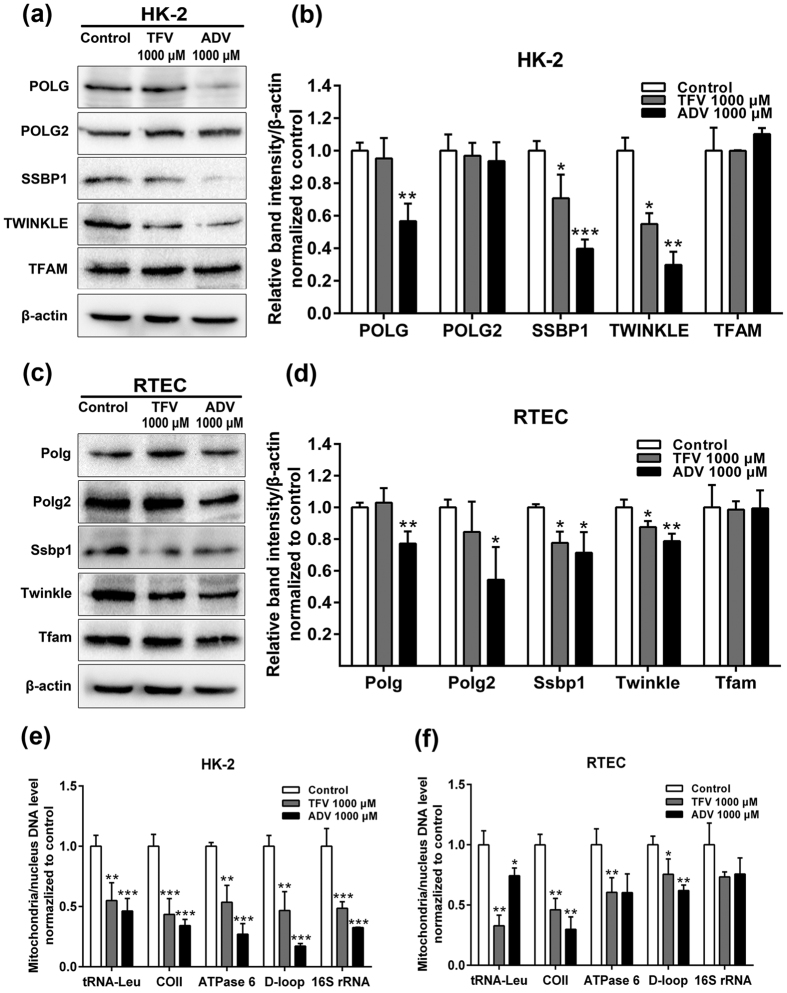
Analysis of genes involved in mtDNA replication after TFV and ADV treatments. (**a**) Western blot analysis of proteins responsible for mtDNA replication: POLG, SSBP1, TWINKLE, TFAM with β-actin as internal control in whole cell lysates (20 μg protein) obtained from HK-2 cells treated with 1000 μM TFV and ADV. (**b**) Quantification of proteins/β-actin normalized to controls. (**c**) Western blot and (**d**) quantitative analysis for the levels of Polg, Ssbp1, Twinkle and Tfam in RTEC. (**e**) mtDNA levels by real-time PCR for the mtDNA encoded tRNA-Leu, COII, ATPase 6, D-loop and 16 S rRNA in HK-2 cells and (**f**) RTEC. Mitochondria DNA level/nuclear β-globin were normalized to control. The cropped blots are displayed and full-length blots are presented in Supplementary Fig. 2. Values are presented as means ± SEM (N = 3, *P < 0.05, **P < 0.01, ***P < 0.001 vs control).

Mitochondria DNA is replicated by an assembly of proteins in a replisome consisting of DNA polymerase γ (POLG), SSBP1, mitochondrial DNA helicase (TWINKLE), mitochondrial transcription factor A (TFAM) and RNaseH activities^27^. SSBP1, TWINKLE (a direct gene target of SSBP1), TFAM and POLG were down-regulated in our proteomics analysis, pointing to a potential role in the mitochondrial dysfunction observed with TFV and ADV treatment. To validate the mitochondrial DNA replication protein expression changes identified in our proteomics experiment, we quantified protein expression of SSBP1, POLG, TWINKLE and TFAM in HK-2 and RTEC following 5-day treatment with TFV and ADV. POLG but not POLG2 was significantly decreased in ADV treated HK-2 cells (Fig. 4a,b) although both POLG and POLG2 protein expression were reduced with ADV treatment of RTEC (Fig. 4c,d). SSBP1 and its downstream gene target TWINKLE were significantly decreased in both HK-2 and RTEC cultures (1000 μM) (Fig. 4a–d), consistent with the proteomic data (Fig. 3f). TFAM had no significant differences compared with the control groups (Fig. 4a–d).

tRNA-Leu, ATP synthase subunit 6 (ATPase 6), Cytochrome c oxidase subunit 2 (COII), D-loop and 16 S rRNA are the major components of mtDNA^28^. mtDNA levels were measured by Real-time PCR (RT-PCR) to quantify the amount of mtDNA relative to nuclear DNA^28^. The mtDNA levels of representative mitochondrial genes (tRNA-Leu, ATPase 6, COII and D-loop) decreased in both HK-2 cells and RTEC treated with TFV and ADV (1000 μM) (Fig. 4e,f). 16S rRNA level was only significantly changed in HK-2 cells treated with TFV and ADV (1000 μM) (Fig. 4e). We found that overexpression of SSBP1 significantly improved HK-2 cell viability and mtDNA gene expression levels following TFV and ADV treatments (Supplementary Fig. 3c,d). Collectively this data indicates that mitochondrial dysfunction following TFV and ADV treatments is due to decreased expression of mtDNA replicating genes like SSBP1 and PLOG^17^,^18^.

### TFV and ADV metabolically reprogram glucose metabolism through down-regulating TRAP1

**Figure 5:**
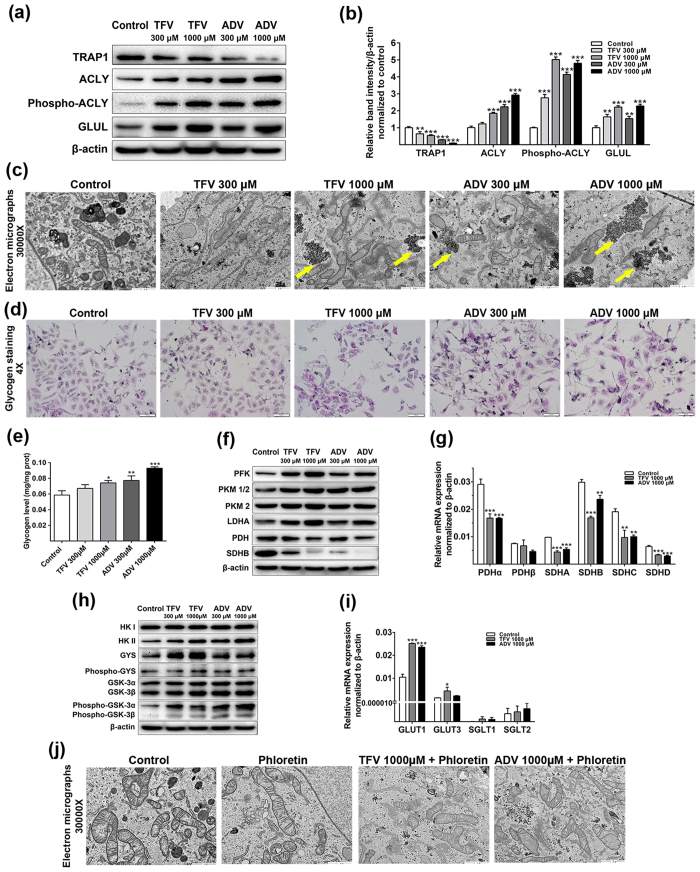
Analysis of glucose and glycogen pathways mediated by TFV and ADV treatment in HK-2 cells. (**a**) Western blot analysis of prominent proteins from proteomic data: TRAP1, ACLY, phospho-ACLY and GLUL with β-actin as internal control. (**b**) Quantification of proteins/β-actin above, normalized to controls. (**c**) Transmission electron microscopy (TEM) analysis of ultrastructure in HK-2 cells. Glycogen is visualized as small dark granules (yellow arrows) (original magnification × 30000, marker indicates 1 μm). (**d**) Periodic Acid Schiff (PAS) staining of glycogen in HK-2 cells treated with or without TFV and ADV. The magnification is 4× and the scale bar represents 100 μm. (**e**) Glycogen quantification. Glycogen levels were normalized by cells protein concentration measured by the BCA assay. (**f**) Western blotting analysis of PFK, PKM1/2, PKM2, LDHA, PDH, SDHB in glucose pathway, using β-actin as internal control. (**g**) Real-time PCR for mRNA expression of multiple subunits of PDH and SDH. (**h**) Western blotting analysis of HK, GYS, phosphor-GYS, GSK-3α/β, phosphor-GSK-3α/β in glycogen pathway. (**i**) Real-time PCR for mRNA expression of glucose transporters GLUT1, 3 and SGLT1, 2. (**j**) Glycogen detection in HK-2 treated with glucose transporter inhibitor phloretin by TEM. The cropped blots are displayed and full-length blots are presented in Supplementary Fig. 2. Values are presented as means ± SEM (N = 3, *P < 0.05, **P < 0.01, ***P < 0.001 vs control).

Our proteomic analysis identified TRAP1 as the most down-regulated protein following TFV and ADV treatment and was therefore selected for further study for a potential role in TFV and ADV induced renal toxicity. Consistent with the proteomic analysis, TRAP1 protein levels were reduced in both treatments compared with untreated group as determined by immunoblot analysis (Fig. 5a). Furthermore, ACLY and GLUL were significantly elevated in both TFV and ADV treated HK-2 cells (1000 μM) (Fig. 5a,b), including increased phosphorylated ACLY.

Altered expression of proteins required for glucose homeostasis suggests this process may be disrupted with TFV and ADV treatment. TEM cellular ultrastructure analysis displayed significant glycogen accumulation, seen as small dark granules (yellow arrows) in the cytoplasm of HK-2 treated with TFV and ADV (Fig. 5c, Supplementary Fig. 2c). PAS staining of glycogen revealed a significant increase of total cellular glycogen compared with the controls (Fig. 5d, Supplementary Fig. 2d). Further, glycogen quantification showed significant accumulation in intracellular glycogen in HK-2 treated with TFV (1000 μM) and ADV (300 μM and 1000 μM), indicating that glucose metabolism was disturbed in the cells treated by TFV and ADV (Fig. 5e).

With these observations on TRAP1 (glucose metabolism modulator) and glycogen accumulation by TFV and ADV treatments, we examined the protein expression of key enzymes regulating glucose and glycogen metabolism. In the glycolysis pathway, glycolysis pathway proteins phosphofructokinase (PFK), pyruvate kinase (PKM) but not PKM2 and lactate dehydrogenase A (LDHA) were up-regulated in a dose-dependent manner in HK-2 cells after TFV and ADV treatments (Fig. 5f, Supplementary Fig. 4a). In contrast, TCA cycle proteins pyruvate dehydrogenase (PDH) and succinate dehydrogenase subunit B (SDHB) were down-regulated following treatments with TFV or ADV (Fig. 5f, Supplementary Fig. 4a). Gene expression of PDH and SDH subunit genes were also significantly down-regulated following TFV or ADV treatment (Fig. 5g).

We next examined expression of enzymes that regulate glycogen metabolism. Hexokinase II (HK II), glycogen synthase (GYS) and phosphorylation of glycogen synthase kinase 3α/β (Phospho-GSK-3α/β) were significantly up-regulated after both treatments whereas HK I, phospho-GYS and GSK-3α/β showed no appreciable difference compared with controls (Fig. 5h, Supplementary Fig. 4b). RT-PCR of relevant genes in glucose pathways revealed that glucose transporters (GLUT1 and 3) were significantly increased but mRNA expression levels of renal glucose transporter (SGLT1 and 2) were low and not significantly affected by TFV and ADV treatment (Fig. 5i). TRAP1 overexpression in HK-2 cells significantly decreased transcript expression of GLUT1 and HK II and blocked TFV and ADV-induced up-regulations of GLUT1 and HK II expression (Supplemental Fig. 3f).

Increased expression of glucose-metabolizing enzymes suggested that the glucose metabolism was potentially reprogramed to favor glycogen formation. To further explore how glucose uptake affects the glycogen accumulation by TFV and ADV treatments, we co-treated HK-2 cells with TFV or ADV and the glucose transporter inhibitor phloretin. Glycogen accumulation (small dark granules in EM images) observed in HK-2 cells treated TFV and ADV (Fig. 5c), were significantly reduced when cells were co-treated with phloretin (Fig. 5j, Supplementary Fig. 2c).

### *In vivo* studies of mice treated with TDF and DPADV

**Figure 6:**
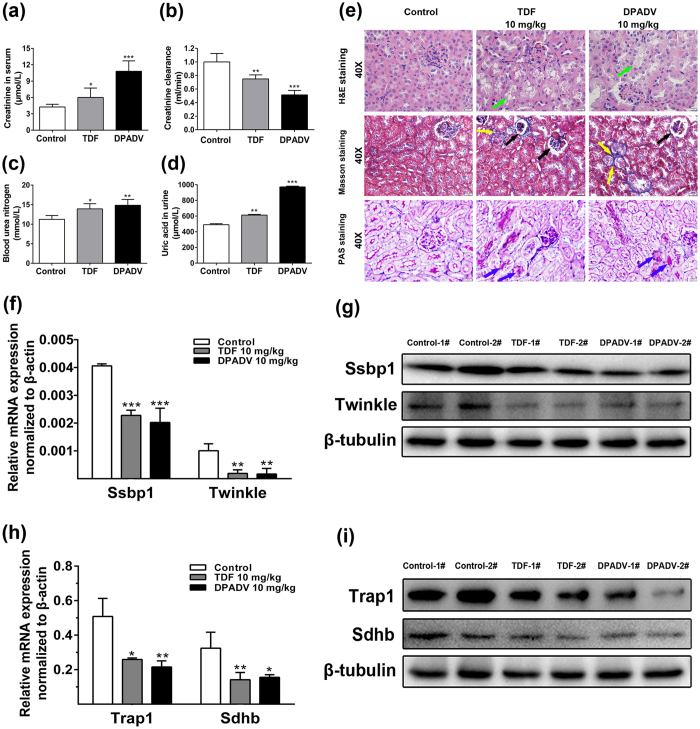
Effects of TDF and DPADV administration for 16-week on mouse kidney. (**a**) Creatinine levels and (**b**) creatinine clearance calculation in serum of mice after TDF and DPADV treatments. (**c**) Blood urea nitrogen and (**d**) uric acid levels were detected in urine. (**e**) Representative histopathology of kidney tissue was assessed by H&E, masson’s trichrome and PAS staining. Tubular vacuolization (green arrows), anilin blue fibrosis (yellow arrows) and dark purple brush border loss and abnormal glycogen aggregation (blue arrows) were shown. The magnification is 40× and the scale bar represents 20 μm. (**f**) Real-time PCR for mRNA expression of Ssbp1 and Twinkle in kidney of mice. (**g**) Western blot analysis of Ssbp1 and Twinkle with β-tubulin as internal control. (**h**) Real-time PCR and (**i**) western blot analysis for Trap1 and Sdhb levels. The cropped blots are displayed and full-length blots are presented in Supplementary Fig. 2. Values are presented as means ± SEM (N = 5, *P < 0.05, **P < 0.01, ***P < 0.001 vs control).

To further explore the effects of TFV and ADV treatments on renal toxicity and evaluate the molecular pathways we described above *in vivo*, we treated mice with TDF and DPADV at 10 mg/kg for 8 weeks. After that, serum and urine were analyzed for kidney functional tests. First, we examine the level of blood urea nitrogen (BUN), creatinine and uric acid as the markers for kidney function^29^. The mean creatinine levels in serum were significantly increased (Fig. 6a) and creatinine clearance was decreased (Fig. 6b). BUN and uric acid in urine were higher in TDF, DPADV treatments compared with control group (Fig. 6c,d), indicating TDF and DPADV treatments lead to kidney dysfunction in mice.

We used histology of mouse kidney (H&E, Masson’s trichrome and PAS staining) to examine renal injuries stemming from TDF and DPADV treatment. Kidneys of mice treated by TDF and DPADV had loss of tubular cells and tubular vacuolization (green arrows) as compared with control mice (Fig. 6e, top panels). Masson’s trichrome staining disclosed higher interstitial fibrosis (yellow arrows), shrunken glomerular tufts and glomerular sclerosis (black arrows) in TDF and DPADV treated mouse kidney (Fig. 6e, middle panels). PAS staining revealed brush border loss (blue arrows) and abnormal glycogen accumulations in TDF and DPADV mice compared with control kidney (Fig. 6e, bottom panels). Quantification of kidney histology is shown in Supplementary Fig. 2e–h.

We next tested protein expression of mtDNA replication machinery in the mouse kidneys treated with TDF and DPADV. Similar to HK-2 cells, Ssbp1 and Twinkle expression decreased significantly in the kidneys of TDF and DPADV treated mice (Fig. 6f,g, Supplementary Fig. 4c). Key regulators of glucose metabolism Trap1 and Sdhb were also reduced in the TDF and DPADV treated mice (Fig. 6h,i, Supplementary Fig. 4d). The protein level reductions of TRAP1 and SSBP1 *in vivo* were not to the same degree as observed in human tubular HK-2 cells and mouse RTEC cells. The discrepancy might result from the differences of drug accumulations in targeting protein between the whole kidney and cultured tubular cells (HK-2 and RTEC) that are likely the main sites of TFV and ADV action.

## Discussion

**Figure 7:**
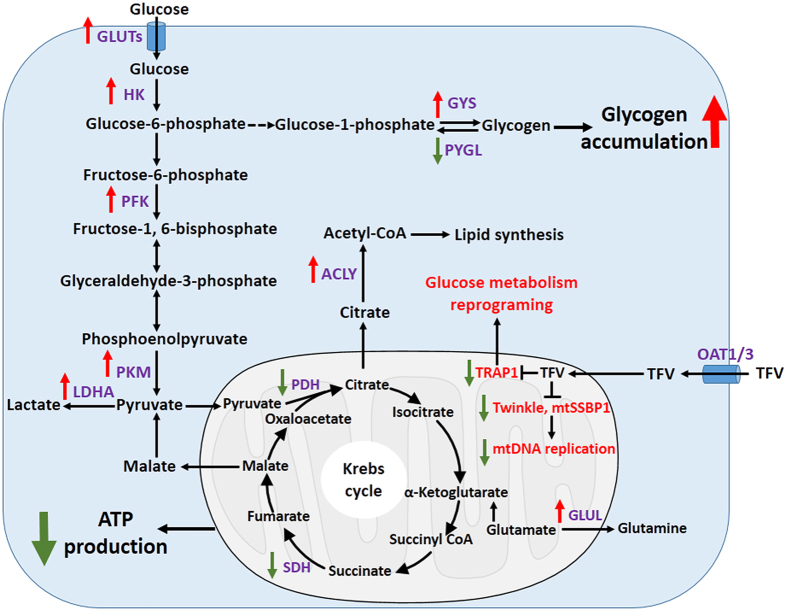
Proposed mechanism of TFV and ADV induced renal toxicity in the proximal tubule (PT) cells. TFV and ADV is taken up from blood into PT cells via basolateral membrane OAT1/3 and is subsequently secreted from PT cells into urine through apical membrane efflux MRP4. Due to abnormal transport or other genetic factors, accumulated TFV concentrations inside PT cells will reduce mtDNA level by inhibiting SSBP1 and TWINKLE protein responsible for mtDNA replication. mtDNA is involved in many oxidative phosphorylation and damage of mtDNA induces disruption of ATP production. Among the genes regulated by TFV and ADV treatments, mitochondrial chaperone TRAP1 may play a central role in reprograming glucose metabolism by inhibiting its glucose oxidative phosphorylation and increasing glycolysis and glycogen synthesis. That is, down-regulation of PDHα and SDHB in TCA cycle and increasing protein levels of GLUT1, HK, PFK, PKM and GYS in glycolysis and glycogen synthesis. Elevation of bioenergetic genes ACLY and GLUL may help cells to generate energy from other sources like glutamine or lipid. Increased glycogen accumulation may be toxic for renal tubule cell function.

Various nephrotoxic side effects have become increasingly problematic and scrutinized despite the therapeutic success of TFV and ADV in treatment of HIV and HBV infections. Emerging evidences point to metabolic dysfunction in the kidney with abnormal glycogen and lipid metabolism likely playing critical roles in renal disease development and drug toxicity^30^,^31^. In this study, we have shown that mitochondriopathy resulting from TFV and ADV ‘rewires’ the glucose metabolism and results in abnormal glycogen accumulation. We propose that this may be a causative factor for nephrotoxicity seen with TFV and ADV therapy. The overall proposed mechanism and pathways are summarized in Fig. 7.

Acute tubular necrosis or dysfunction with TFV and ADV treatment has been long speculated to be caused by reduced activity of mtDNA POLG^16^. Our proteomic data and western blot validation revealed that mtDNA replicating gene SSBP1, along with TWINKLE and POLG were significantly down-regulated (Fig. 4a, Supplementary Table 2)^27^,^32^,^33^, indicating the mtDNA replication machinery were less active after TFV and ADV treatments. Possibly, SSBP1 could be the main regulated gene in mtDNA replication complex by TFV and ADV treatments. Of note, our proteomic data revealed other significantly altered proteins not related to mtDNA replication and further studies are needed to explore their roles in renal toxicity by TFV and ADV treatments.

TRAP1 plays important roles in glucose metabolism, cell survival and cytoprotection function^34^,^35^. Previous work has shown that TRAP1 deletion reprograms the glucose oxidative phosphorylation and glycolysis causing increased ATP production and glucose metabolism^36^,^37^,^38^. TRAP1-deficiency reduces glycolysis in murine adult fibroblast whereas it promotes glycolysis in hepatocyte and mouse embryonic fibroblast^38^. We believe that decreased TRAP1 expression is important for understanding how TFV and ADV induce renal toxicity. TRAP1 down-regulation by TFV and ADV treatment may potentially rewire the glucose metabolism by fueling more glucose to glycolysis and glycogen synthesis but less to oxidative phosphorylation because of mitochondria dysfunction. Alternatively, TRAP1′s down-regulation by TFV and ADV might be cellular survival mechanism to cope with the ATP shortfall due to mitochondrial injury.

Our analysis of key enzymes within TCA cycle identified that the expression of SDHB was substantially reduced in TFV and ADV treated HK-2 cells compared with the controls (Fig. 5). SDHB suppression provides more evidence of damaged energy production from oxidative phosphorylation. TRAP1 interacting with SDHB in the context of mitochondrial bioenergetics is complex^24^. Our observations are consistent with previous reports that the deficiency of TRAP1 increases glycolysis in the cytosol (Figs 2d and 5f)^24^,^39^,^40^. Moreover, we showed that overexpression of TRAP1 resulted in significant down-regulation GLUT1 and HK II, required for glucose uptake and metabolism. The reduction of TRAP1 may further up-regulate pyruvate kinase and lactate dehydrogenase through aerobic glycolysis to produce ATP and cope with the energy shortfall^24^. However, the oxidative phosphorylation is still reduced as mitochondria was damaged by TFV and ADV treatments, which may be the major site of oxidative phosphorylation. The expression decreases of different subunits in SDH and PDH of TCA cycle following TFV and ADV treatments in HK-2 cells indicate that down-regulation of TRAP1 profoundly affected the respiratory chain to reduce oxidative phosphorylation (Fig. 5g). Our evidence pointing to glucose reprogram by TFV and ADV could be helpful to understand rare incidences of fatal lactic acidosis in patients during treatment with tenofovir^41^.

Glycogen accumulation in TFV and ADV treated HK-2 cells is another piece of evidence that glucose metabolism may be metabolically reprogrammed via TRAP1. The up-regulation of PFK expression might increase the glucose consumption through glycolysis^42^,^43^ (Fig. 2d). On the other hand, it might not consume all of extra glucose from increased glucose uptake (Fig. 5i,j) and down-regulation of TCA cycle (Figs 2b and 5g), which could go to the glycogen pathway (Fig. 5h). This observation is particularly intriguing as it provides a novel mechanism of TFV and ADV induced renal toxicity. The accumulation and deposition of glycogen in renal tubular cells leads to Armani-Ebstein lesions and acute kidney failure^44^,^45^. We propose that TFV and ADV exposure may reprogram the metabolic status of the cells, feeding more glucose to glycogen synthesis (Fig. 7) as the glucose oxidative phosphorylation and the mitochondria damage proceeds. It is possibly that up-regulation glucose transporter GLUT1 and GLUT3 by TFV and ADV treatments in HK-2 cells are due to attempts by the cell to use more glucose for ATP production to counteract mitochondrial dysfunction. Though our analysis is based on TFV and ADV treatment in preclinical models, examining patients with long-term TFV treatment for evidence of disrupted glucose metabolism and glycogen accumulation in renal tubular cells may be an important next step in identifying their molecular mechanisms of renal toxicity and possibly preventing nephrotoxicity caused by use of these medications.

In summary, we present systematic approaches to dissect the molecular mechanism of TFV and ADV induced renal tubular toxicity. Our results demonstrate that renal tubular mitochondriopathy caused by TFV and ADV treatment results from two events: 1) inhibiting mtDNA replicating complex through SSBP1 and 2) down-regulating mitochondrial chaperone TRAP1 and SDHB to rewire the glucose metabolism. The consequent glycogen accumulation represents a previously unidentified mechanism for TFV and ADV associated renal toxicity.

## Methods

### Chemicals and reagents

TFV and ADV were purchased by Sigma-Aldrich Biotechnology (St. Louis, MO, USA); TDF and DPADV were purchased from Selleck Chemicals (Houston, TX, USA); Annexin V-FITC Apoptosis Detection Kit was obtained from KeyGEN Biotech. Co., Ltd (Nanjing, China); Mitochondrial membrane potential assay kit was from Beyotime (Nanjing, China); XF Cell Mito and Glycolysis Stress Test Kits were from Seahorse Bioscience (North Billerica, MA, USA); Hematoxylin and eosin (H&E), Masson’s trichrome and Periodic acid Schiff (PAS) kits were from Solarbio; 3-(4, 5-dimethylthiazol-2-yl)-2, 5-diphenyltetrazolium bromide (MTT) and sulforhodamine B (SRB) were purchased from Sigma.

### Animals

C57BL/6J mice (4 weeks old) were obtained from Vital River (Beijing, China). The mice were randomly assigned to the TDF group, the DPADV group and vehicle-only control group with n = 6 each. The mice were given daily gavage administration of 0.2 ml Sodium carboxymethylcellulose (CMC) containing 10 mg/kg/day TDF and DPADV for 8 weeks. Dosing was carried out by daily gavage at doses that resemble human therapy on a mg/kg/d basis. Patients receive 300 mg/d TDF tablets (Gilead Sciences, CA, USA). All animal surgery was performed under anesthesia by 4% chloral hydrate, and anesthetized animals were sacrificed by cervical dislocation at the end of the experiments. All experiments were performed in accordance with guidelines of the Institute for Laboratory Animal Research of Tsinghua University. The experimental procedures were approved by the Administrative Committee of Experimental Animal Care and Use of Tsinghua University, licensed by the Science and Technology Commission of Beijing Municipality (SYXK-2014-0024), and they conformed to the National Institute of Health guidelines on the ethical use of animals.

### Cell culture

HK-2 were purchased from ATCC (CRL-2190, Manassas, VA, USA). HK-2 cells were cultured in Dulbecco’s Modified Eagle’s Medium. RTEC were harvested from mice kidney and the cortices of kidney were dissected, minced, and digested by collagenase type I from Gibco (Grand Island, NY, USA). Fragments of the tissues were filtered with 100 mesh filter. Then the deposition was dissociated by gradient centrifugation with 45% percoll from Sigma. The isolate was implanted in the cell culture dishes. The bred cells were sub-cultured and identified as RTEC by immunocytochemistry and transmission electron microscopy. RTEC were cultured with the same reagents as described above.

To overexpress human SSBP1 and TRAP1, the whole coding sequences of SSBP1 (NM_001256510.1) and TRAP1 (NM_016292.2) were amplified and cloned into pcDNA3.0 between KpnI and XhoI. The forward primer of SSBP1 was 5′-GTACGGTACCATGTTTCGAAGACCTGTATTACAG-3′, and the reverse primer was 5′-CAGACTCGAGCTACTCCTTCTCTTTCGTCTGG-3′. The forward primer of TRAP1 was 5′-GTACGGTACCATGGCGCGCGAGCTGCGGGCGCTG-3′, and the reverse primer was 5′-CAGACTCGAGTCAGTGCGCTCCAGGGCCTTG-3′. The eukaryotic expression vectors pcDNA3.0-SSBP1 and pcDNA3.0-TRAP1 were constructed. HK-2 cells were transfected with pcDNA3.0-SSBP1, pcDNA3.0-TRAP1 and pcDNA3.0 vector as empty control using the Lipofectamine^TM^ 2000 transfection reagent (Invitrogen, Camarillo, CA, USA) according to the manufacturer’s instructions, and cultured for 48 h. Thereafter, G418 was added at the final concentration of 800 μg/ml for 2 weeks and until antibiotic-resistant colonies were observed for the stable SSBP1 and TRAP1-overexpressed HK-2 cell clone.

### Cytotoxicity Assay with IncuCyte^TM^ ZOOM

HK-2 cells were seeded in a 96-well plate and treated with TFV and ADV (0, 30, 300, 1000, 1500 μM) for 5 days. YOYO^®^-1 fluorescently stains the nuclear DNA that have lost plasma membrane integrity. Cells were placed in an IncuCyte™ ZOOM (Essen BioScience, Ann Arbor, MI, USA). Two images per well were collected every 3 hours in both phase contrast and fluorescence. Cells were incubated to allow nuclear DNA staining by YOYO^®^-1. Apoptosis rates were determined by flow cytometry (BD Biosciences, San Jose, CA, USA). HK-2 cells were treated with TFV and ADV (0, 300, 1000 μM) for 5 days. Cells were incubated with Annexin V-FITC and propidium iodide (PI) (KeyGEN Biotech, Nanjing, China) for apoptosis^46^.

### Cell viability assay

HK-2 and RTEC were seeded into 96-well plates and exposed to TFV and ADV (0, 30, 300, 500, 1000 μM) for 5 days. Cells were incubated with MTT for 4 h and the absorbance was detected at 570 nm. For SRB assay, cells were treated as described above then fixed *in situ* by adding to each well cold trichloroacetic acid (10%, w/v) and incubating for 60 min. SRB solution was added to each well and the plates were incubated for 10 min. Bound stain was solubilized with unbuffered tris base (pH 10.5), and the optical densities were read at 492 nm.

### Mitochondria membrane potential assays

Briefly, HK-2 cells were loaded with JC-1 at 37 °C for 20 min, and then analyzed by an Olympus fluorescent microscope (Tokyo, Japan). JC-1 monomer is green, whereas the membrane potential of energized mitochondria promotes the formation of red-fluorescent JC-1 aggregates. Mitochondrial permeability transition was assessed by amount of red fluorescence in each treatment condition, which indicates the presence of mitochondria with a lower membrane potential (ΔΨ_m_). Fluorescence images were collected every 30 seconds by fluorescence excitation/emission maxima: 514/529 nm, monomer form; 585/590 nm J-aggregate form for the duration of the 30 min experiment.

### Mitochondrial and glycolysis stress test assays

HK-2 cells were seeded in 96-well Seahorse XF^e^-96 assay plates and treated with TFV and ADV (300, 1000 μM) for 48 hours. Then, cells were washed and changed to unbuffered DMEM media and incubated for 1 hour. Oxygen consumption rate, was automatically calculated and recorded by mitochondria stress test using the Seahorse XF^e^-96 software (Seahorse Bioscience, North Billerica, MA, USA). The respiratory control ratio (RCR) was determined by using 2 μM oligomycin. The maximal respiration was measured with 0.5 μM FCCP. The cells were then treated with 0.5 μM antimycin A/rotenone, inhibitor of complex III, in order to measure the non-mitochondrial respiration. Measurements were taken after each addition of mitochondrial inhibitor before injection of the next inhibitor. For extracellular acidification rate (ECAR) was measured by glycolysis stress test. Non-glycolytic acidification was defined as initial and final ECARs. Glycolysis was defined as ECAR after injection of 10 mM D-glucose and maximum glycolytic capacity was calculated following addition of 2 μM oligomycin. At last 50 mM 2-DG was injected to inhibit glycolysis.

### ATP determination assay

HK-2 cells were seeded into 6-well plates and exposed to TFV and ADV (0, 300, 1000 μM) for 5 days. Cells were washed with PBS twice, lysed with CelLytic^TM^ (C2978, Sigma, USA) and centrifuged to collect cell supernatant. The quantity of ATP was measured by using an ATP determination kit (A22066, Molecular Probes, USA) following the manufacturer’s instructions. Luminescence was measured using an EnSpire™ Multimode Plate Reader (PerkinElmer, MA, USA). Cells protein concentrations were determined using a BCA assay. ATP production was expressed as μM/μg cells protein.

### Examination of ultrastructural changes

HK-2 cells treated with TFV and ADV (0, 300, 1000 μM) for 5 days were grown on dishes containing glass cover slips to subconfluency and chemically fixed with 2% glutaraldehyde buffered insodium cacodylate buffer. Cells were harvested, washed with cacodylate buffer, and embedded in 2% agarose. Staining was performed with 1% osmium tetroxide and 1% uranyl acetate/1% phosphotungstic acid. Dehydration of samples was done using graded acetone series. Specimens were embedded in spurr epoxy resin and incubated for polymerization at 65 °C for 24 h. Sections were inspected with a transmission electron microscope (H-7650, Hitachi, Japan). The area with glycogen granules accumulation in every image was computed to quantify the glycogen levels. More than 20 images were scored in each condition.

### PAS staining and glycogen quantification

HK-2 cells following treatment with TFV and ADV (0, 300, 1000 μM) for 5 days were washed, fixed in PAS fixative solution and then stained with PAS for subsequent examination under a light microscope (Olympus, Tokyo, Japan). The PAS yielded signal was captured in full colour using bright field. Images were converted to greyscale and the mean optical density of the PAS-derived signal was semiquantified per individual HK-2 cell. More than 100 cells were scored per experiment. An image of not PAS-staining without glycogen was captured to correct background optical density. The amount of glycogen from HK-2 cells treated with TFV and ADV was determined with a Glycogen Assay Kit (Solarbio). Glycogen levels were normalized by cells protein concentration measured by the BCA assay.

### Western blot

Sample were lysed with radioimmunoprecipitation assay (RIPA) buffer containing Halt Protease/Phosphatase inhibitors (Thermo Fisher Scientific, Pittsburgh, PA, USA). The antibodies as follows: anti-Hexokinase I (2024), anti-Hexokinase II (2867), anti-LDHA (3582), anti-PKM1/2 (3190), anti-PKM2 (4053), anti-PDH (3205), anti-PFKP (8164), anti-Glycogen synthase (3893), anti-phospho-Glycogen synthase (3891), anti-phospho-GSK-3α/3β (9327), anti-GSK-3α/3β (5676), anti-ACL (4332), anti-phospho-ACL (4331), anti-rabbit IgG (7074), anti-mouse IgG (7076), Cell Signaling Technology; anti-POLG (ab128899), anti-Glutamine synthetase (ab178422), Abcam; anti-POLG2 (10997-2-AP), anti-SSBP1 (12212-1-AP), anti-Twinkle (13435-1-AP), anti-TFAM (9998-1-AP), anti-β-actin (66009-1-1g), Proteintech Group Inc.; anti-TRAP1 (GTX102017), Genetex; anti-SDHB (YT5450), Immunoway; anti-β-Tubulin (BE0025), Easybio.

### Real-time PCR analysis

Total RNA was extracted by RNAprep pure kit (DP430 and DP431) and DNA was extracted by TIANamp genomic DNA kit (DP304) purchased from Tiangen (Beijing, China). Reverse transcription was performed using TIANScript RT kit (KR104-02). All RT-PCR reactions were carried out on ABI ViiA™ 7 Real-Time System (Life Technologies) using TransStart Top Green qPCR SuperMix (AQ131-03) from Transgen (Beijing, China). All of the PCRs were performed in triplicate, and the specificity of the PCR products was confirmed using melting curve analyses. The housekeeping genes GAPDH and β-actin were used as an internal control.

### Histological examinations of kidney sections

Mice were anesthetized 8 weeks after treatment with TDF and DPADV (10 mg/kg). Kidneys were dissected and fixed in 4% paraformaldehyde solution. The kidney segments were dehydrated in serial alcohol solutions. Tissues were then embedded in paraffin, cut into 5-μm-thick sections, stained with H&E, Masson’s trichrome and PAS examined under a light microscope (Olympus). All H&E, Masson’s trichrome and PAS staining sections were evaluated for injury by an experienced pathologist (VDD) who was blinded to the treatment each animal had received. Renal tubular injury was quantified by determining the number of injured tubules per 200 total tubules.

### Biochemical markers of serum and urine

Mice were maintained under fasting condition for 24 h and urine was collected using a metabolic cage. A cardiac puncture was performed to obtain blood and serum was separated by centrifugation. The harvested urine and serum were immediately stored at −80 °C for subsequent analysis. The supernatant of serum and urine above were analyzed for serum creatinine (sCr), blood urea nitrogen (BUN) and uric acid using a fully automatic biochemical analyzer (Toshiba, Tokyo, Japan).

### Metabolomics

After treatment with TFV and ADV (0, 1000 μM) for 5 days, HK-2 were added by 80% methanol and then incubated at −80 °C for 3 h. Cells were harvested and isolated by centrifugation at 14,000 g for 20 min at 4 °C. The protein concentration of the pellet was measured by the BCA assay for normalization. The metabolite-containing supernatant of cells was dried under nitrogen flow for subsequent analysis. The UPLC system was coupled to a Q-Exactive plus orbitrap mass spectrometer (Thermo Fisher Scientific). Extracts were separated by an ACQUITY UPLC BEH Amide column. The supernatant from the sample was loaded to normal phase chromatography column, and eluted to orbitrap mass spectrometer. The stationary phase was 95% acetonitrile with 5 mM ammonium acetate. Data with mass ranges of m/z 76–1125 and m/z 80-1200 was acquired at positive and negative ion mode with data dependent MSMS acquisition. The full scan and fragment spectra were collected with resolution of 70,000 and 17,500 respectively. Metabolite identification was based on Tracefinder search with home-built database, with a MS1 mass error of <8 ppm and MS2 mass error of <15 ppm. Data were analyzed by searching metabolic databases, including Kyoto Encyclopedia of Genes and Genomes (KEGG), Human Metabolome Database (HMDB) and METLIN. The peak area list with compound names was analyzed by R scripts revised from the source code of MetaboAnalyst 3.0^47^,^48^. The positive and negative dataset were transformed by generalized log and Pareto Normalization respectively to make different compounds comparable.

### Proteomics

Following TFV and ADV treatment (0, 1000 μM) for 5 days, HK-2 were washed and added with 500 μl 8 M urea. Cells were scraped and transferred to a 1.5 ml tube, incubated at 4 °C for 30 min, centrifuged for 10 min and protein concentrations were determined using a BCA assay. Two hundred micrograms of proteins were reduced with 1 mM dithiotreitoland alkylated with 5.5 mM iodoacetamide. Proteins were digested with trypsin for overnight, and stopped by 10% trifluoracetic acid. The peptides were desalted using C18 sep-pak cartridges and eluted with 1 ml methanol. Peptides were redissolved in Tetraethylammonium Bromide and labeled using TMT sixplex labeling reagent. The TMT-labeled peptides were combined and desalted by C18 sep-pak cartridges. The fractions were centrifuged and analyzed by LC-MS/MS. The TMT-labeled peptides were separated by gradient elution in a Thermo-Dionex Ultimate 3000 HPLC system. The analytical column was a home-made C18 resin packed fused silica capillary column. The Q Exactive mass spectrometer was operated by Xcalibur 2.1.2 software and 10 data-dependent MS/MS scans followed a single full-scan mass spectrum in the orbitrap. The peak lists from LC-MS/MS analysis were generated with Proteome Discoverer software. The MS/MS spectra were searched by the human FASTA database. Peptide spectral matches were validated using the Percolator at a 1% false discovery rate. The false discovery rate was set to 0.01 for protein identifications. Relative protein quantification was performed by Proteome Discoverer software. Protein ratios were calculated as the median of all peptide hits belonging to a protein. Quantitative precision was expressed as protein ratio variability. The biological meaning of proteomic data is first analyzed by Gene Ontology analysis and KEGG pathway enrichment. And we using SIGNOR database to generate a literature-based signaling information^49^,^50^. The knowledge based data obtained from STRING10 database is used to analyze the association of the common differently expressed proteins. Cytoscape 3.4.0 was used to visualize the common, differently expressed proteins.

### Statistical analysis

Statistical analyses were performed using GraphPad Prism software, version 6.0. Values were presented as mean ± SEM. This analysis was performed for three independent experiments at least. Unpaired t-tests were performed for comparison between two groups. Data were analyzed using a one-way analysis of variance (ANOVA) followed by a Newman–Keuls multiple comparison test. Statistical significances were calculated and indicated. (***P < 0.001, **P < 0.01 and *P < 0.05).

## Additional Information

**How to cite this article**: Zhao, X. *et al*. Tenofovir and adefovir down-regulate mitochondrial chaperone TRAP1 and succinate dehydrogenase subunit B to metabolically reprogram glucose metabolism and induce nephrotoxicity. *Sci. Rep.* **7**, 46344; doi: 10.1038/srep46344 (2017).

**Publisher's note:** Springer Nature remains neutral with regard to jurisdictional claims in published maps and institutional affiliations.

## Supplementary Material

Supplementary Information
