# Supplementary material for: Tenofovir and adefovir down-regulate mitochondrial chaperone TRAP1 and succinate dehydrogenase subunit B to metabolically reprogram glucose metabolism and induce nephrotoxicity
Source: Sci Rep. 2017 Apr 11;7:46344. doi: 10.1038/srep46344 (PMC5387747; doi:10.1038/srep46344)
Supplement: Supplementary Information [file srep46344-s1.pdf]

Tenofovir and adefovir down-regulate mitochondrial chaperone TRAP1 and succinate dehydrogenase subunit B to metabolically reprogram glucose metabolism and induce nephrotoxicity

Xinbin Zhao[1], Kun Sun[1], Zhou Lan[1], Wenxin Song[1], Lili Cheng[1], Wenna Chi[1,2], Jing Chen[1], Yi Huo[3], Lina Xu[4], Xiaohui Liu[4], Haiteng Deng[3], Julie A. Siegenthaler[5], Ligong Chen[1,2]*

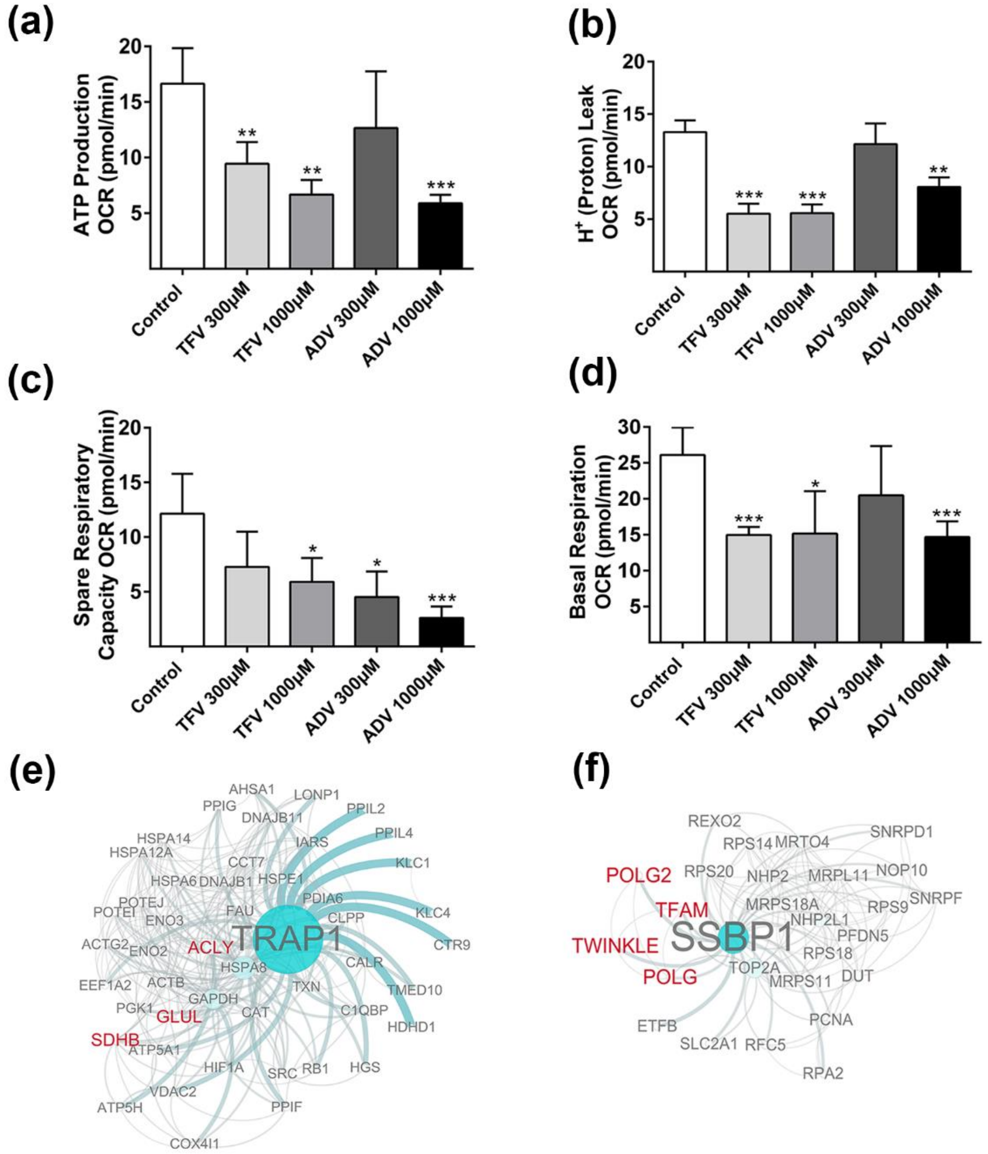

Supplementary Figure 1. Quantitative analysis of mitochondria and glycolytic function by seahorse assay. (**a**) ATP production, (**b**) H+ proton leak, (**c**) spare respiration and (**d**) basal respiration were calculated from the mean OCRs by mitochondria stress test. **(e)** Analysis of protein interactions and signaling pathway of TRAP1. (**f**) Analysis of protein interactions and signaling pathway of the regulation of SSBP1. Values are presented as means ± SEM (N=3, *P<0.05, **P<0.01, ***P<0.001 vs control).

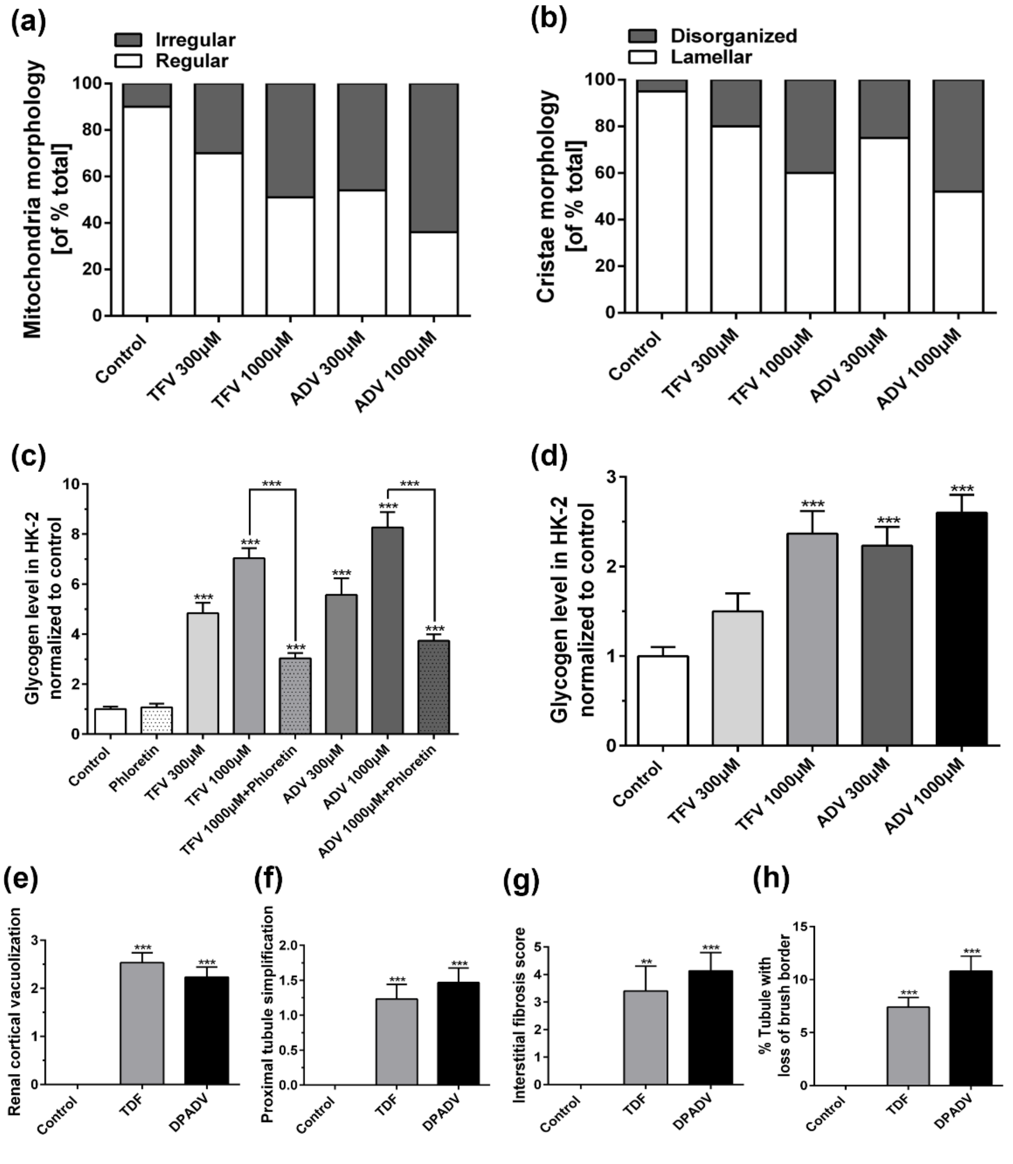

Supplementary Figure 2. Quantitative analysis. (**a**) Quantification of mitochondria morphology in HK-2 treated with TFV and ADV. Cells containing regular shapes (white bars) and irregular shapes including large/small rings, elongated/aggregated/fragmented ones (black bars) were classified. More than 100 cells were scored per experiment. (**b**) Quantification of cristae morphology in HK-2 exposed to TFV and ADV. More than 30 cells and approximately 10 sections of individual cells were scored per experiment. (**c**) Quantification of glycogen granules from transmission electron microscopy images. The area with glycogen granules accumulation in every image was computed to quantify the glycogen levels. More than 20 images were scored in each condition. (**d**) Quantification of glycogen levels from images of HK-2 stained by PAS. More than 100 cells were scored per experiment. Quantification of (**e**) renal cortical vacuolization and (**f**) proximal tubule simplification for kidney sections from mice treated with TDF and DPADV (10 mg/kg). (**g**) Quantitative kidney interstitial fibrosis score (Masson's trichrome staining). (**h**) Quantitative loss of PAS-positive tubular brush borders. Values are presented as means ± SEM (*P<0.05, **P<0.01, ***P<0.001 vs control)

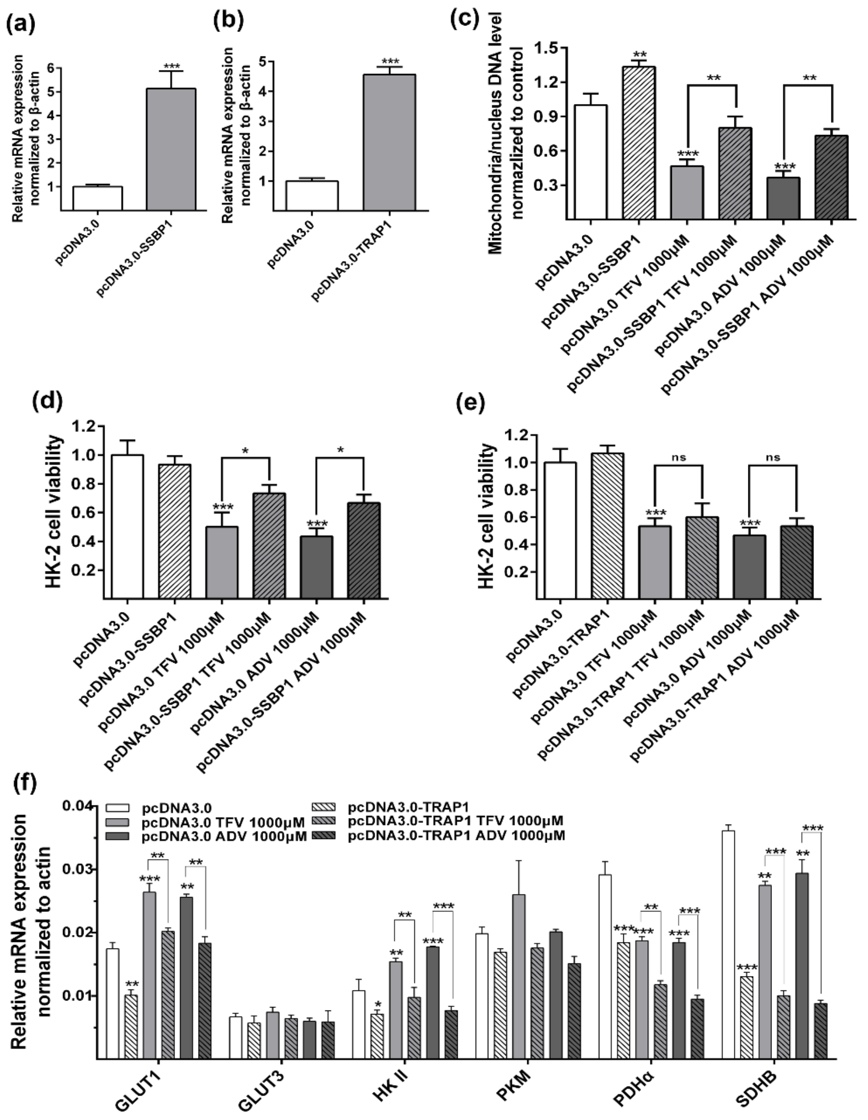

Supplementary Figure 3. Effects of TFV and ADV in SSBP1 and TRAP1 overexpressed HK-2 on cell toxicity and metabolism. (**a, b**) Real-time PCR for mRNA expression of SSBP1 and TRAP1 in HK-2 overexpression cell lines. (**c**) mtDNA levels by real-time PCR for the mtDNA encoded D-loop after TFV and ADV treatment. Mitochondria DNA level/nuclear β-globin were normalized to control. Cell viability was determined by MTT assay in (**d**) SSBP1 and (**e**) TRAP1 overexpressed HK-2 cells after TFV and ADV. (**f**) Real-time PCR for mRNA expression of GLUT1, GLUT3, HKII, PKM, PDH and SDHB using β-actin as internal control in HK-2 overexpression of SSBP1 and TRAP1.

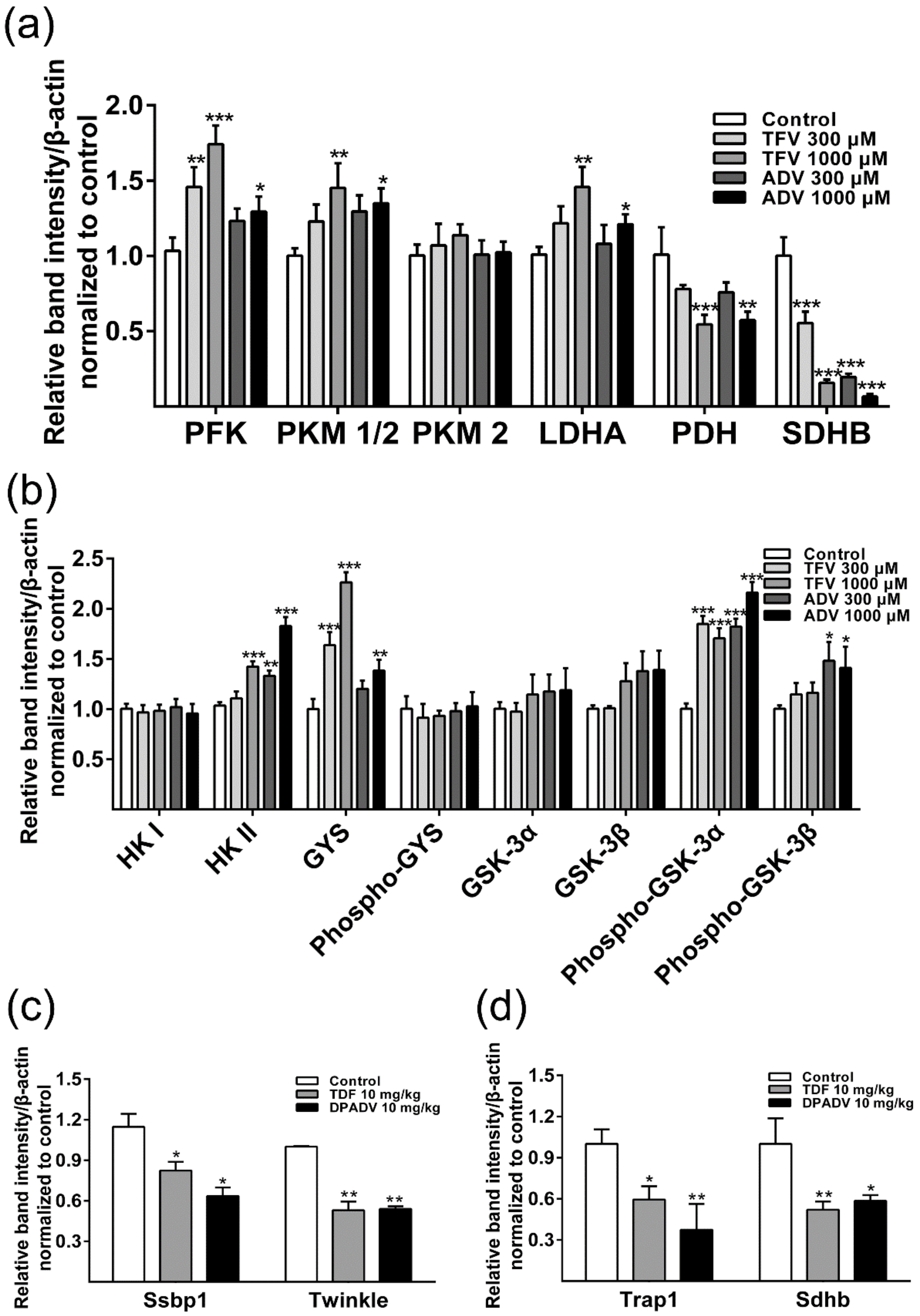

Supplementary Figure 4. Quantitative analysis of proteins from western blot. Quantification of these proteins: (**a**) PFK, PKM1/2, PKM2, LDHA, PDH, SDHB in HK-2; (**b**) HK, GYS, phosphor-GYS, GSK-3α/β, phosphor-GSK-3α/β in HK-2 with β-actin as internal control normalized to control; (**c**) Ssbp1 and twinkle and (**d**) trap1 and sdhb in kidney with β-tubulin.

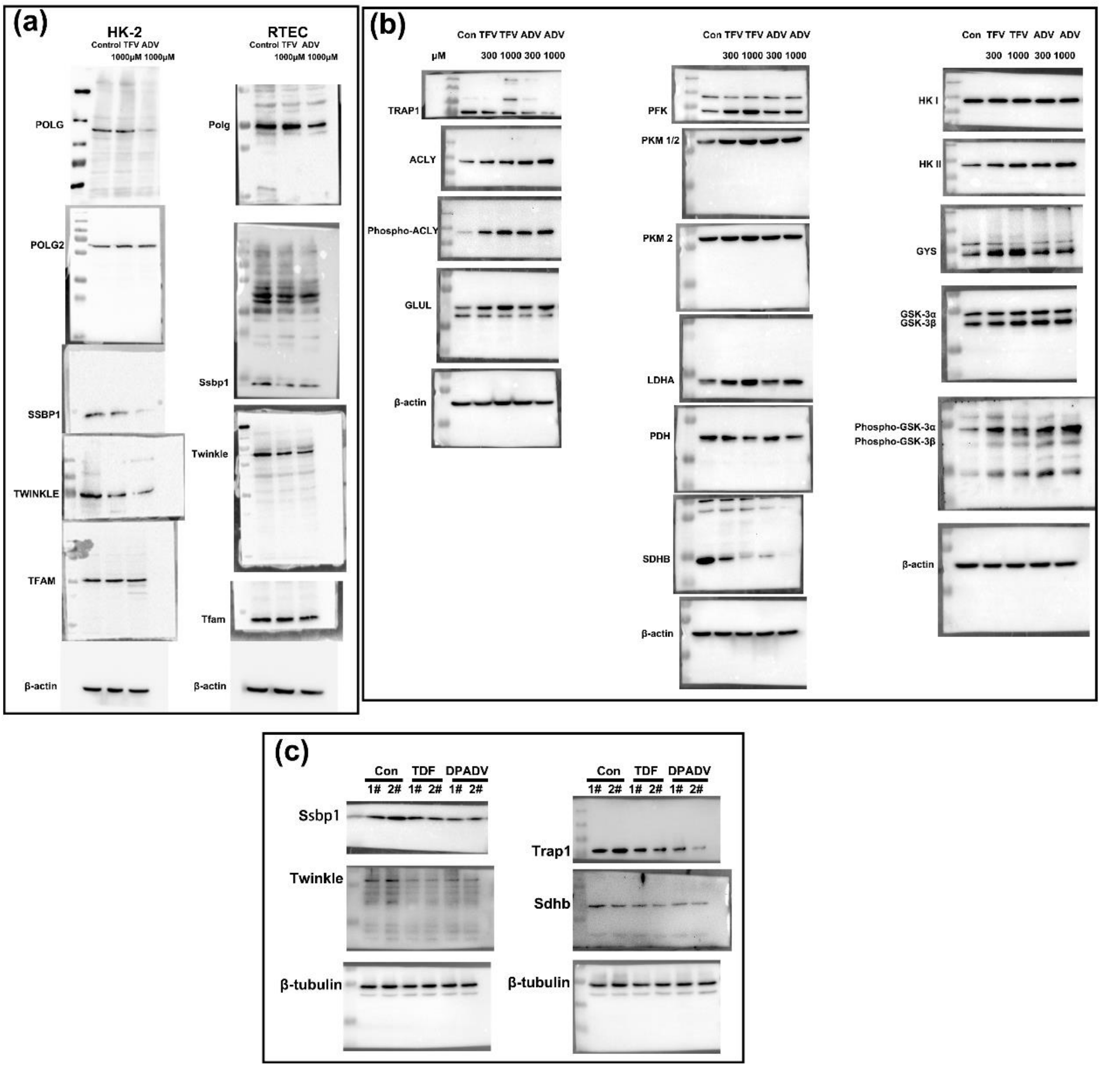

Supplementary Figure 5. The display of uncropped western blots. Full-length of these proteins: (**a**) POLG, SSBP1,TWINKLE, TFAM in HK-2; (**b**) TRAP1, ACLY, Phospho-ACLY, GLUL, PFK, PKM1/2, PKM2, LDHA, PDH, SDHB, HK, GYS, GSK-3α/β, phosphor-GSK-3α/β in HK-2 with β-actin; (**c**) Ssbp1, twinkle, trap1 and sdhb in kidney with β-tubulin.

Table 1 Changes of metabolites detected in HK-2 treated with TFV and ADV (1000 μM)

Metabolite	Extracted Mass	Retention time (min)	Mass m/z	Ratio TFV/Con	p-value	Ratio ADV/Con	p-value
ADP	426.02	11.36	-0.25	2.500	0.0049101	2.356	0.0043589
AMP	348.07	10.31	-1.02	1.896	0.0088568	1.856	0.0020959
Acetylcarnitine	204.12	7.72	-1.12	0.062	0.0012816	0.237	0.0015650
Adenine	134.05	4.32	-0.06	0.049	0.0005903	0.027	0.0009160
5'-Methylthioadenosine	298.10	2.54	-1.77	0.552	0.0029317	0.675	0.0486713
Adenosine	268.10	4.69	-1.69	0.093	0.0000733	0.171	0.0000774
Betaine	189.16	12.12	-2.21	0.273	0.0002639	0.479	0.0002967
Choline	104.11	5.90	-1.50	1.995	0.0000095	2.352	0.0009919
Citrulline	176.10	9.69	-2.28	0.144	0.0004903	0.139	0.0004175
Creatine	132.08	8.68	-2.76	0.093	0.0006221	0.271	0.0005871
Cytarabine	244.09	6.43	-2.49	0.004	0.0017377	0.058	0.0020225
Cytosine	112.05	5.41	-1.53	2.482	0.0025280	2.756	0.0033956
Deoxycytidine	228.10	5.60	-2.46	0.072	0.0018792	0.101	0.0018417
D-Glutamylglycine	205.08	10.60	-2.27	2.835	0.0004692	2.565	0.0003771
Gluconic acid	195.05	9.42	-0.57	0.077	0.0017985	0.194	0.0019217
Glycerophosphocholine	258.11	9.42	-1.75	0.003	0.0021693	0.047	0.0024505
Hexanoylcarnitine	260.19	5.82	-1.85	0.713	0.0124030	0.461	0.0002075
Hydroxyphenyllactic acid	181.05	6.03	-0.43	0.062	0.0016339	0.081	0.0013302
Hypoxanthine	137.05	4.60	-3.78	3.530	0.0026159	9.247	0.0010497
Iditol	183.09	6.57	5.38	0.064	0.0004846	0.120	0.0006145
Imidazoleacetic acid	127.05	2.23	-3.12	1.724	0.0031957	1.761	0.0031445
L-Acetylcarnitine	204.12	7.72	-1.12	0.249	0.0000340	0.196	0.0000299
L-Valine	118.09	7.63	-1.75	0.273	0.0002639	0.479	0.0002967
Methacholine	160.13	9.48	-3.28	0.124	0.0018169	0.389	0.0037390
N1-Acetylspermidine	188.18	14.29	-2.62	4.990	0.0002020	4.492	0.0005272
Niacinamide (Low)	123.06	1.86	-2.73	2.339	0.0280231	1.927	0.0175201
Oleamide	282.28	4.10	-1.83	2.279	0.0034561	2.271	0.0026915
Phosphocreatine	212.04	11.18	-2.69	0.443	0.0032146	0.431	0.0028964
Proline	116.07	7.82	-1.96	0.263	0.0020258	0.335	0.0030688
Propionylcarnitine	218.14	7.11	-2.07	0.046	0.0001567	0.116	0.0000528
Spermine	203.22	7.33	-2.77	0.597	0.0000470	0.612	0.0005658
Taurine	126.02	7.80	-2.23	0.009	0.0018176	0.113	0.0023947
Thiamine	265.11	7.63	-1.17	0.278	0.0014804	0.508	0.0044785
Thymidine	243.10	4.41	-0.76	0.223	0.0031269	0.035	0.0015595
Thymine	127.05	2.23	-3.12	1.724	0.0031957	1.761	0.0031445
Uric acid	167.02	11.96	-0.44	0.056	0.0040348	0.042	0.0040348

Supplementary Table 1. Changes of metabolites detected in HK-2 cells treated with TFV and ADV. Comprehensive profile of metabolites were identified by a Q-Exactive plus orbitrap mass spectrometer equipped with a heated electrospray ionization probe. Data were collected for peak extraction and quantification of ion intensities and analyzed by searching metabolic databases, including KEGG, HMDB and METLIN for putative matches to known metabolites. The dataset were transformed by generalized log and Pareto Normalization respectively to make different compounds comparable.

Table 2 Quantitative analysis of proteins treated with TFV and ADV in HK-2 cells

Protein_accession	Protein names	Gene symbol	Score	Coverage	# Unique peptides	# PSMs	Ratio TFV/Con	Ratio ADV/Con
**Housekeeping proteins**								
P60709	Actin, cytoplasmic 1	ACTB	2659.29	85.60	1	895	1.087	1.293
Q9BQE3	Tubulin alpha-1C chain	TUBA1C	910.73	72.16	17	299	1.027	1.044
P07437	Tubulin beta chain	TUBB	1028.27	86.49	8	381	1.037	1.099
K7EIS0	Tubulin gamma chain (Fragment)	TUBG1	4.06	6.79	1	1	1.059	1.016
**ATP synthase proteins**								
P30049	ATP synthase subunit delta, mitochondrial	ATP5D	12.34	22.02	4	5	0.820	0.707
H7BXK9	ATP-binding cassette sub-family B member 6, mitochondrial	ABCB6	0.00	2.9	1	1	0.879	0.875
P18859	ATP synthase-coupling factor 6, mitochondrial	ATP5J	11.16	23.15	2	3	0.806	0.738
P36542	ATP synthase subunit gamma, mitochondrial	ATP5C1	9.26	14.43	3	3	0.912	0.930
**Mitochondria DNA replication and mitochondria related proteins**								
I3L0K7	Heat shock protein 75 kDa, mitochondrial	TRAP1	34.89	6.26	1	14	0.491	0.323
Q04837	Single-stranded DNA-binding protein, mitochondrial	SSBP1	14.34	43.24	3	4	0.665	0.500
F5H1D6	DNA polymerase	POLE	6.29	3.76	6	7	0.933	0.955
Q00059-2	Isoform 2 of Transcription factor A, mitochondrial	TFAM	2.66	4.67	1	1	0.861	0.986
Q5JTZ9	Alanine--tRNA ligase, mitochondrial	AARS2	2.78	2.23	1	1	0.753	0.761
P14406	Cytochrome c oxidase subunit 7A2, mitochondrial	COX7A2	1.62	15.66	1	1	0.866	0.776
O43615	Mitochondrial import inner membrane translocase subunit TIM44	TIM44	6.35	7.74	3	3	0.867	0.885
Q3ZCQ8	Mitochondrial import inner membrane translocase subunit TIM50	TIMM50	9.69	15.86	3	3	0.847	0.836
P30084	Enoyl-CoA hydratase, mitochondrial	ECHS1	84.11	44.83	12	26	0.860	0.849
O75380	NADH dehydrogenase [ubiquinone] iron-sulfur protein 6, mitochondrial	NDUFS6	5.52	19.35	1	1	0.748	0.724
Q5QPE7	Mitochondrial genome maintenance exonuclease 1	MGME1	9.07	0.952865	1	2	0.806	0.953
Q15785	Mitochondrial import receptor subunit TOM34	TOMM34	49.16	1.1022335	9	13	1.001	1.102
**Apoptosis related proteins**								
C9JLV4	Apoptotic protease-activating factor 1	APAF1	9.78	4.64	3	3	1.137	1.155
S4R3H4	Apoptotic chromatin condensation inducer in the nucleus	ACIN1	100.61	27.44	31	37	1.053	0.975
Q5SVL2	Caspase-7 (Fragment)	CASP7	5.09	18.47	2	2	1.101	1.149
Q10570	Cleavage and polyadenylation specificity factor subunit 1	CPSF1	22.51	4.71	4	6	1.014	0.985
Q8N163	Cell cycle and apoptosis regulator protein 2	CCAR2	54.35	25.24	16	20	1.019	0.951
Q9H8G2	Caspase activity and apoptosis inhibitor 1	CAAP1	7.71	11.91	2	4	1.022	0.919
O43293	Death-associated protein kinase 3	DAPK3	2.40	10.57	4	5	1.205	1.113
O14737	Programmed cell death protein 5	PDCD5	76.46	78.4	17	29	1.013	0.946
Q12888	Tumor suppressor p53-binding protein 1	TP53BP1	32.74	10.4	11	13	1.084	1.020
Q03169	Tumor necrosis factor alpha-induced protein 2	TNFAIP2	23.02	15.29	8	9	1.005	1.176
Q9NYF8-2	Isoform 2 of Bcl-2-associated transcription factor 1	BCLAF1	50.37	15.9	14	25	1.070	1.058
Q13625-2	Isoform 2 of Apoptosis-stimulating of p53 protein 2	TP53BP2	12.15	5.77	4	4	1.110	1.017
O95831-3	Isoform 3 of Apoptosis-inducing factor 1, mitochondrial	AIFM1	49.04	30.05	13	16	0.977	1.061
Q07812-5	Isoform Epsilon of Apoptosis regulator BAX	BAX	5.11	28.66	4	5	1.058	1.039
Q14249	Endonuclease G, mitochondrial	ENDOG	7.84	10.1	2	3	0.940	0.918
P62820	Ras-related protein Rab-1A	RAB1A	37.33	48.78	4	13	0.919	0.902
Q9BTC0	Death-inducer obliterator 1	DIDO1	28.66	11.79	13	13	1.035	0.879
Q16531	DNA damage-binding protein 1	DDB1	28.19	10.44	8	10	1.033	0.818
Q9NUG6	p53 and DNA damage-regulated protein 1	PDRG1	9.31	12.78	1	3	0.833	0.589
D6RHU3	Caspase-6 subunit p18 (Fragment)	CASP6	0.00	8.39	1	1	1.130	0.934
**Others**								
P53396-2	Isoform 2 of ATP-citrate synthase	ACLY	290.93	46.65	1	94	1.911	1.360
P53396	ATP-citrate synthase	ACLY	303.75	46.68	2	98	1.267	1.227
P15104	Glutamine synthetase	GLUL	11.03	4.29	1	3	1.227	1.636
P01023	Alpha-2-macroglobulin	A2M	6.22	1.56	2	2	1.693	1.742
O43747	AP-1 complex subunit gamma-1	AP1G1	11.35	4.38	3	4	1.309	1.377
P02765	Alpha-2-HS-glycoprotein	AHSG	11.30	7.08	3	5	2.022	1.850
P51397	Death-associated protein 1	DAPK3	4.34	20.59	2	2	1.349	1.670
Q96J02-2	Isoform 2 of E3 ubiquitin-protein ligase Itchy homolog	ITCH	3.91	4.06	3	3	1.611	1.485
Q8TAE8	Growth arrest and DNA damage-inducible proteins-interacting protein 1	GADD45GIP1	0.00	10.81	2	2	0.877	0.769
Q00839	Heterogeneous nuclear ribonucleoprotein U	HNRNPU	285.11	48	3	97	1.535	1.772
H0YMT9	Annexin (Fragment)	ANXA2	97.40	80.45	1	36	0.775	0.644

Supplementary Table 2. Quantitative analysis of proteins treated with TFV and ADV in HK-2 cells. Relative protein quantification was performed by Proteome Discoverer software and proteins containing two unique peptides at least were regarded as confident identifications. Protein ratios were calculated as the median of all peptide hits belonging to a protein. Quantitative precision was expressed as protein ratio variability. The mass spectrometry proteomics data have been deposited to the ProteomeXchange consortium. The biological meaning of proteomic data is firstly analyzed by Gene Ontology analysis and KEGG pathway enrichment. And we using SIGNOR database to generate a literature-based signaling information.
